# Implementing a cancer survivorship seminar course to non-healthcare professional undergraduate students

**DOI:** 10.1007/s00520-024-08426-1

**Published:** 2024-03-13

**Authors:** Alexandre Chan

**Affiliations:** Department of Clinical Pharmacy Practice, School of Pharmacy & Pharmaceutical Sciences, University of California, Irvine, 802 W Peltason Drive, Berk Hall, Irvine, CA 92697-4625 USA

**Keywords:** Undergraduate, Cancer education, Cancer survivorship, KAP

## Abstract

**Background:**

At University of California, Irvine (UCI), a seminar course focused on cancer survivorship was developed and offered to non-healthcare professional undergraduate students. Utilizing the knowledge, attitude, and perception (KAP) framework, this study was designed to examine the impact on students who have taken this course, and to clarify the value of this course for undergraduate students.

**Methods:**

This was a cross-sectional survey. Undergraduate students enrolled in the Life After Cancer Freshmen Seminar course (Uni Stu 3) at UCI between 2021 and 2023 were invited to participate. The survey consisted of 4 main sections: (1) demographics, (2) knowledge of cancer survivorship, (3) attitude towards cancer survivorship, and (4) perception and awareness of cancer survivorship. The survey was administered prior to the implementation of the course, and the same survey was administered at the end of the course.

**Results:**

A total of 33 students completed the pre-implementation survey and 30 students completed the post-implementation survey. Comparing pre- and post-course implementation, there was an increase of perception and awareness of (i) resources and guidelines for cancer survivors (pre, 9.1% vs. post, 36.7%), (ii) mental health complications among cancer survivors (pre, 36.4% vs. post, 56.7%), (iii) benefits of cancer survivorship care (pre, 15.2% vs. post, 40%), latest research in cancer survivorship (pre, 0% vs. post, 23.3%), and (iv) tailoring survivors’ needs according to their age groups (pre, 24.2% vs. post, 66.7%). Knowledge and attitude towards caring of cancer survivors were similar comparing pre- and post-course implementation.

**Conclusion:**

In an undergraduate seminar course focused on cancer survivorship, we observed an improvement of non-healthcare students’ perception and awareness of cancer survivorship-related issues, advocating the value on introducing highly prevalent cancer survivorship topics early to both undergraduate STEM and non-STEM students.

## Background

As of 2022, it is estimated that there are 18.1 million cancer survivors in the United States of America. This represents approximately 5.4% of the population. The number of cancer survivors is expected to increase to 22.5 million by 2032 [1]. The drastic increase of cancer survivors is primarily driven by the advancement of cancer diagnostics, cancer therapeutics, and earlier screening and detection of cancers. With such a growing number of people living with cancer, cancer is evolving into a chronic disease which causes a longer and deeper impact not only on patients but also to caregivers and families.

In view that cancer is more likely to affect older individuals, most young adults do not have extensive experience in interacting with cancer patients or managing complications associated with cancer. Within the United States, much of the cancer education within higher education is focused on prevention of skin cancer [2, 3] or human papillomavirus [4, 5]. Currently, didactic education on cancer is also primarily focused on healthcare students, with most educational program designed for medical [6–9] and nursing students [10–13]. These educational programs are mostly introduced as part of the undergraduate medical and nursing curriculums, and these programs are located outside of the United States. Most of these courses aim to equip the workforce about cancer care. In summary, general concepts on cancer care are not routinely taught within an undergraduate curriculum in the United States.

Currently, there is literature to show the value of teaching science and healthcare topics to non-healthcare professional undergraduates [14–17]. However, it is unknown how undergraduate students perceive a didactic course that is dedicated to cancer survivorship. There is also a lack of literature on the perception of cancer survivorship and its education among undergraduate students.

At University of California, Irvine (UCI), an undergraduate seminar course focused on cancer survivorship was developed and offered annually between 2021 and 2023. Utilizing the knowledge, attitude, and practice/perception (KAP) framework [18], this study was designed to examine the impact of this course on undergraduate students. This study was also designed to clarify the value of such a course offered to undergraduate students.

## Methods

### Study design and setting

This was a cross-sectional survey conducted between 2021 and 2023 at UCI. Founded in 1965, UCI was ranked among the United States’ top 10 public universities by U.S. News & World Report [19]. With close to 30,000 undergraduates enrolled, UCI was designated as an Asian American and Native American Pacific Islander-serving institution. This study was exempted by UCI Investigational Review Board, and a waiver of informed consent to participate was obtained for this study.

### Inclusion/exclusion criteria

Undergraduate students enrolled in the Life After Cancer Freshmen Seminar course (Uni Stu 3) at UCI between 2021 and 2023 were invited to participate in this study.

### Teaching pedagogy behind the freshmen seminars series and Life After Cancer

At UCI, the Freshman Seminar Series is designed to bring a high-impact learning experience to undergraduate students in their first year of study. In a small class environment, students can explore and learn about a special theme or topic by engaging with their peers and the faculty instructor. These seminars are typically scheduled for 1 h a week per quarter, with stimulating discussions and critical thinking being the primary goals. Most seminars are open to all interested students, with no pre-requisites and with enrollment preference given to freshmen. Each seminar course is designed as a one-unit small group seminar enrolling 15 students. Students normally took this course for a letter grade, though students may elect the pass/not pass option.

Designed as one of Freshmen Seminar Series by the principal investigator of this study, Life After Cancer was a 1-unit weekly seminar series designed to introduce concepts of cancer survivorship to undergraduate students. Through a total of 11 weeks of seminars, discussions, and group presentations, students learned how cancer has become a chronic condition in many survivors, especially among those who are cured. Students learned about the long-term complications of cancer treatments, as well as the cutting-edge research that is currently undertaking around the globe to mitigate these complications. The specific learning objectives of the seminar course were to:

1. Understand the definition and issues surrounding cancer survivorship.
2. Identify common toxicities and complications that are affecting various groups of cancer survivors.
3. Appreciate the disease trajectory of common cancers, from diagnosis to survivorship.
4. Discuss management strategies that are commonly employed to manage complications of cancer during survivorship.
5. Discuss the impact of cancer survivorship on the health care system.
6. Discuss the research directions that are taken to address the concerns related to cancer survivorship.

In this course, a number of topics were taught including the following: (1) an orientation of cancer management and cancer survivorship; (2) trajectory of cancer treatment from diagnosis to cancer survivorship; (3) survivorship in the elderly; (4) survivorship in pediatric, adolescent, and young adults; (5) survivorship in stem cell transplant patients; (6) model of survivorship care and rehabilitation technology.

When the course was offered in 2021, there  were two assignments for this course, which included a group presentation and a term paper. However, the assignment was reduced to the group presentation only in 2022 and 2023.

- For the group presentation, students were assigned in groups to present a 15-min PowerPoint presentation with 10 min Q&A on the management of a toxicity that is commonly faced by cancer survivors. Two students were randomly paired to present on one of the following issues: cardiotoxicity, cognitive impairment, fatigue, financial toxicity, infertility, pain, and peripheral neuropathy. Students were required to use evidence-based information to introduce the management strategies.
- For the term paper, each student was assigned to write a 1500-word research paper on an assigned survivorship-related topic.

The seminar course was offered and taught for three consecutive years between 2021 and 2023. When the course first launched in January 2021, the course was taught virtually in view of the COVID-19 pandemic. The course was then subsequently taught fully in person in April 2022 and April 2023.

### Data collection

During the first 2 weeks of the course, a pre-implementation survey was administered to the students. The survey was administered electronically using Qualtrics® in 2021 as the course was taught virtually, while the survey was administered on paper in 2022 and 2023. At the end of the course, the same survey was administered to the students before the end of the course.

### Survey instrument

In view of the lack of a validated tool available for this study, a survey instrument was designed by the principal investigator (A.C.) of the study after conducting an extensive literature search on the impact of medical education on undergraduate students [3, 4, 7–13, 15]. As part of the survey development process, a panel of experts in cancer survivorship (R.C., Y.K., C.J.T., Y.L.T., D.Q.N.) reviewed the survey and provided feedback on the user-friendliness and appropriateness of the questions to the principal investigator. The survey instrument consisted of 4 main sections: (1) demographics, (2) knowledge of cancer survivorship, (3) attitude towards cancer survivorship, and (4) perception and awareness of cancer survivorship.

#### Knowledge items

Respondents were asked to determine whether each of the 10 items was true or false. Three items were related to general understanding of cancer survivorship, five items focused on outcomes/toxicities issues among cancer survivors, and two items focused on lifestyle issues in cancer survivorship. These knowledge items were derived from learning objectives of each weekly seminar.

#### Attitude items

Respondents were asked to rate each statement using a 4-level Likert scale (strongly disagree, disagree, agree, and strongly agree). Three items focused on respondents’ comfort level in listening and responding to concerns of a cancer survivor, as well as looking after own family member who is a cancer survivor. Three items focused on respondents’ attitude on education, whether it was appropriate to offer the course to college freshmen, respondents’ understanding of cancer as a chronic disease, and respondents’ interest in pursuing a healthcare profession (general vs cancer specialist).

#### Perception items

Respondents were asked to rate each statement using a 4-level Likert scale (strongly disagree, disagree, agree, and strongly agree). Six items focused on their awareness of current resources, mental health complications, benefits, research, personalization of cancer survivorship, and conceptualizing cancer as a chronic disease.

### Statistical analysis

Descriptive statistics were used to summarize responses to each item. The chi-square test or Student’s *t*-test was conducted for cross-sectional analyses to determine whether demographics were different before and after course implementation, with a two-sided *p* value < 0.05 considered statistically significant. Paired analysis was not conducted in view a few respondents dropped out during the course. All statistical analyses were conducted using SPSS version 28.

## Results

### Demographics

A total of 33 students completed the pre-implementation survey and 30 students completed the post-implementation survey. Ten students enrolled in 2021, 11 in 2022, and 12 in 2023. In the pre-implementation survey, majority of the respondents were female (69.7%), with a mean (± SD) age of 20 ± 1.7 years old. Seventeen respondents (51.5%) were majoring in STEM subjects, with biological sciences (24.2%), undecided (21.2%), and pharmaceutical sciences (15.2%) being the top 3 majors among the respondents. Demographics of respondents during the post-implementation survey were similar to those in the pre-implementation survey (Table 1).

**Table 1:** Demographics of students responded pre- (*n* = 33) and post- (*n* = 30) course implementation

	Pre-implementation	Post-implementation	*p* value
Completed survey (*n*)	33	30	
Gender (*n*, %)			0.75
Male	10, 30.3%	8, 26.7%	
Female	23, 69.7%	22, 73.3%	
Mean age (SD)	20 (1.7)	20 (1.1)	0.40
Mean units (SD)	15 (2.9)	15 (2.5)	0.87
Majors (*n*, %)			0.92
STEM majors	17, 51.5%	17, 56.7%	
Anthropology	1, 3%	1, 3.3%	
Biological Sciences	8, 24.2%	7, 23.3%	
Biomedical Engineering	2, 6.1%	1, 3.3%	
Business Administration	3, 9.1%	3, 10%	
Criminology, Law & Society	0, 0%	1, 3.3%	
Economics	1, 3%	0, 0%	
Environmental Science and Policy	1, 3%	0, 0%	
History	1, 3%	0, 0%	
International Studies	1, 3%	0, 0%	
Mechanical Engineering	1, 3%	2, 6.7%	
Performance Arts	1, 3%	2, 6.7%	
Pharmaceutical Sciences	5, 15.2%	5, 16.7%	
Psychology	0, 0%	1, 3.3%	
Public Health Policy	1, 3%	1, 3.3%	
Sociology	0, 0%	1, 3.3%	
Undecided	7, 21.2%	5, 16.7%	

### Knowledge regarding cancer survivorship

Respondents’ accuracy for each item ranged from 21.2 to 97.0% in the pre-implementation survey. The top 3 items that were most likely to be answered correctly were as follows: “Elderly cancer survivors are at higher risks for heart problems after chemotherapy.” (97.0%), “Chemobrain tends to co-exist with cancer-related fatigue” (90.9%), and “Smoking cessation and weight management are common lifestyle management strategies in cancer survivors.” (90.9%) (Table 2).

**Table 2:** Comparing respondents’ true/false responses on knowledge statements pre-implementation versus post-implementation

	Statement	Answer	Category	Pre-implementation, answered correctly (*n*, %)	Post-implementation, answered correctly (*n*, %)
1	Breast cancer is largely incurable, with over half of the patients initially diagnosed as late stage	False	Disease	26, 78.8%	26, 86.7%
2	Survivorship care plan should be implemented 5 years after cancer diagnosis	False	General concept	22, 66.7%	21, 70%
3	Smoking cessation and weight management are common lifestyle management strategies in cancer survivors	True	Lifestyle	30, 90.9%	27, 90%
4	More than half of the young cancer survivors are able to resume schooling without problems after their cancer treatment	False	Outcomes/toxicities	17, 51.5%	17, 56.7%
5	Bone health should be monitored in the care of prostate and breast cancer survivors	True	Outcomes/toxicities	27, 81.8%	24, 80%
6	Cancer survivors should not receive vaccinations immediately after their chemotherapy because of their elevated risk for infections	False	Lifestyle	7, 21.2%	9, 30%
7	Elderly cancer survivors are at higher risks for heart problems after chemotherapy	True	Outcomes/toxicities	32, 97.0%	25, 83.3%
8	Fear of cancer recurrence is uncommon among cancer survivors	False	Outcomes/toxicities	29, 87.9%	25, 83.3%
9	“Chemobrain” tends to co-exist with cancer-related fatigue	True	Outcomes/toxicities	30, 90.9%	29, 96.7%
10	Current research focuses on the transition of cancer survivors from primary care settings to specialty care settings	False	General concept	8, 24.2%	6, 20%

Respondents’ accuracy for each item ranged from 20.0 to 96.7% in the post-implementation survey. The top 3 items that were most likely to be answered correctly were as follows: “Chemobrain tends to co-exist with cancer-related fatigue” (96.7%), “Smoking cessation and weight management are common lifestyle management strategies in cancer survivors.” (90%), and “Breast cancer is largely incurable, with over half of the patients initially diagnosed as late stage.” (86.7%) (Table 2).

### Attitude towards cancer survivorship

**Fig. 1:**
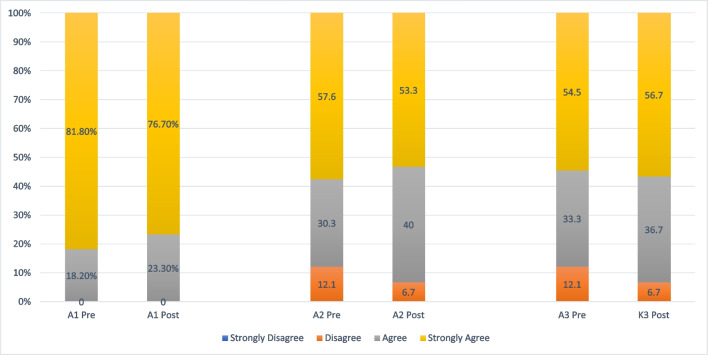
Comparing respondents’ responses pre-implementation (*n* = 33) versus post-implementation (n=30) on attitude for caring cancer survivors. (A1) I am comfortable in listening to the concerns of a cancer survivor; (A2) I am comfortable in responding to the concerns of a cancer survivor; (A3) I am comfortable in looking after my own family member who is a cancer survivor

Most respondents indicated that they were most comfortable listening to cancer survivors’ needs (strongly agree, 81.8%), followed by responding to survivors’ concerns (strongly agree, 57.6%) as well as looking after a family member who is a cancer survivor (strongly agree, 54.5%). (Figure 1) Similar results were observed after implementation of the seminar, with most respondents indicated that they were most comfortable listening to survivors’ needs (strongly agree: 76.7%), responding to their concerns (strongly agree, 53.3%) and looking after a family member who is a cancer survivor (strongly agree, 56.7%).

**Fig. 2:**
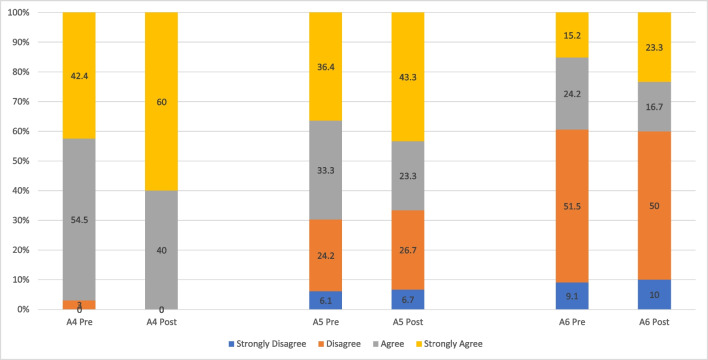
Comparing respondents’ responses pre-implementation (*n* = 33) versus post-implementation (*n* = 30) on education perspectives related to cancer survivorship. (A4) It is appropriate timing to educate concepts of cancer survivorship to university freshmen; (A5) I am interested in pursuing a healthcare profession after my bachelor’s degree; (A6) I am interested in pursuing a healthcare profession after my bachelor’s degree and specialized in taking care of patients diagnosed with cancer

In terms of whether it was appropriate to educate cancer survivorship concepts to freshmen, more respondents strongly agreed after implementation (pre, 42.4% vs. post, 60.0%). Similarly, slightly more respondents strongly agreed that they were interested in pursuing a healthcare profession after their bachelor’s degree (pre, 36.4% vs. post, 43.3%), as well as pursuing a healthcare profession and specialized in taking care of patients diagnosed with cancer (pre, 15.2% vs. post, 23.3%) (Fig. 2).

### Perception towards cancer survivorship

**Fig. 3:**
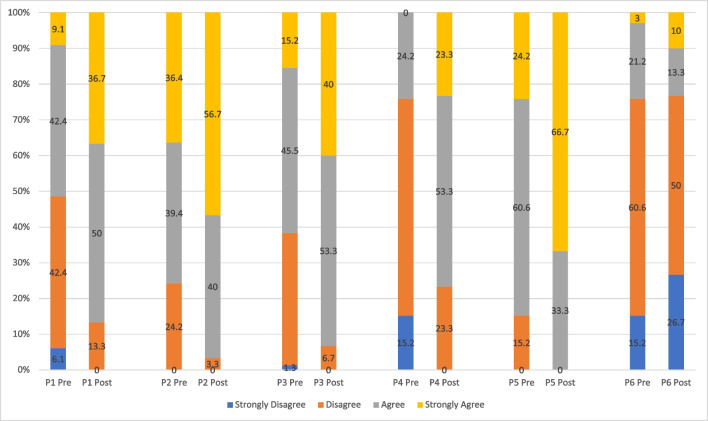
Comparing respondents’ responses pre-implementation (*n* = 33) versus post-implementation (*n* = 30) on their perceptions and awareness related to cancer survivorship. (P1) I am aware of the resources and guidelines available for cancer survivors to seek for information; (P2) I am aware of the mental health complications among cancer survivors; (P3) I am aware of the benefits of cancer survivorship care; (P4) I am aware of the latest research areas in cancer survivorship; (P5) I am aware that the caring of cancer survivors needs to be tailored according to their age groups; (P6) I find it difficult to conceptualize cancer as a chronic disease

Comparing respondents’ perception and awareness before and after course implementation, an increase of strong agreement was observed regarding awareness of resources and guidelines available for cancer survivors to seek for information (pre, 9.1% vs. post, 36.7%), mental health complications among cancer survivors (pre, 36.4% vs. post, 56.7%), benefits of cancer survivorship care (pre, 15.2% vs. post, 40%), latest research in cancer survivorship (pre, 0% vs. 23.3%), and tailoring cancer survivors’ needs according to their age groups (pre, 24.2% vs. post, 66.7%) (Fig. 3).

## Discussion

In this study, we have successfully evaluated the impact of an undergraduate seminar course that was developed to teach basic concepts of cancer survivorship. Using the KAP framework, we have observed a modest improvement in students’ perception and awareness towards cancer survivorship. This study is innovative because currently there is a lack of studies evaluating the impact of such clinically focused courses being taught to non-healthcare professional undergraduates. As cancer survivors become more prevalent in our society [20], there is a growing need to provide a broad overview on cancer management to young adults who are undergoing tertiary education. This study provides insights to the education community on the value to offer clinically inclined courses in the earlier part of undergraduate non-healthcare professional education.

Most notably, positive improvements related to the perception and awareness of cancer survivorship were observed. We speculated that these improvements were likely contributed by the various discussions and activities conducted during the course. For example, the group assignment on management of cancer-related toxicities required students to actively look up clinical trials and guidelines on cancer survivorship, as well as the latest areas in cancer survivorship research. Several seminars were devoted to describing the different challenges encountered by various age groups of cancer survivors (pediatrics vs. adolescent and young adults [21] vs. elderly [22]), which has likely increased awareness of the issues and complications faced by survivors of different age groups and potential disparities [23]. The discussions on physical and psychosocial impacts frequently faced by cancer survivors might have also increased students’ understanding of the mental health complications, as well as the benefit of cancer survivorship care. Overall, the course has successfully increased and improved the perception and awareness of cancer survivorship issues among students. The required assignments have also exposed the students to evidence-based healthcare literature, which may improve student empowerment within the learning process [24].

Although the seminar nature of the course has improved perceptions and awareness of cancer survivorships-related issues, two other aspects were not improved among students enrolled in the course: knowledge and attitude towards caring of cancer survivors. Regarding knowledge level, we did not observe any dramatic changes over time. One may argue that students answered certain knowledge items correctly prior to the course because undergraduate students in general have good test-taking skills and perhaps have intuitive knowledge. For example, most students likely found it sensible for cancer survivors to engage smoking cessation and weight management, hence explaining the high accuracy rates observed. Certain knowledge items, however, may be controversial (e.g., whether cancer survivors should receive vaccinations post-chemotherapy) which could be challenging for non-healthcare professional students to provide accurate responses. Lastly, concepts may not have been discussed in detail in class (e.g., transition of cancer survivors from primary care settings to specialty care settings), hence affecting the accuracy rate.

Additionally, we did not observe a change to the students’ interest to provide care to people diagnosed with cancer before and after the implementation of the course. We observed that students were more comfortable with listening to the concerns of cancer survivors (all agreed), with a small proportion of students disagreed that they were comfortable responding and listening to the specific concerns from cancer survivors (including family members). As this course was designed to introduce concepts of cancer survivorship, including practical and research concepts, the course was not designed to provide empathy training which is an important to trait for anyone to provide survivorship care. Studies have shown that empathy-training is very specific which requires specific training activities [25]. It is also unknown what is the most optimal approach to motivate undergraduate non-healthcare students wanting to provide care to cancer patients. In the nursing literature, it was shown that experiential learning is most crucial to increase confidence and reduce fears among undergraduates nurses to provide care to cancer patients [26]. This is a difficult gap to bridge solely through a seminar course, considering the lack of a dedicated experiential component of the students’ training, needless to say that these students also lacked professional identity. Future courses may want to consider incorporating an experiential component, such as interviewing a cancer survivor, in order to provide students opportunity to reflect and create relevance to the topic.

Designed as an introductory course on cancer survivorship to non-healthcare professional undergraduate students, the course has taken an innovative approach to introduce survivorship concepts. Nonetheless, there were challenges associated with the administration of the course. Firstly, with the limited time given (1 h per week for 11 weeks), it was very ambitious to introduce all vital concepts in detail. At UCI, a 1-unit course is roughly equivalent to 3 h of work per week by the students. Additionally, the course was enrolled by students with very diverse, including non-STEM backgrounds (> 40%) which may require extra effort to fully comprehend some of the topics. These students were also carrying a heavy workload (an average of 15 units per quarter). With these considerations in mind, one may question whether an introductory course on cancer survivorship was appropriate to be offered to undergraduate non-healthcare professional students. Despite these limitations, the course was very successful and well-liked by enrolled students. This course was designed to provide a sufficiently broad overview of the topics, with the aim to help students overcoming the fears to learn about a medical topic. Lastly, the course was also designed to introduce the latest research related to cancer survivorship, with the aim to inspire students to consider future graduate studies in healthcare-related subjects.

There were multiple strengths with our study. Data from three separate cohorts were included, as well as pre- and post-assessments, which allowed the comparison of outcomes over time. The study has also evaluated a few dimensions through our surveys, which provided us with information on which domain student had the most benefit from with our approach. There were also several limitations. In view that a few students dropped the course after completing the pre-implementation survey, it was not possible to have the same number of completed questionnaires for pre- and post-implementation. Additionally, the survey results were anonymized, hence it was not possible to evaluate the paired outcomes. The survey primarily captured quantitative results using the KAP approach, hence it did not capture students’ motivations and feelings on this course. Future studies may consider incorporating qualitative approaches (through focus groups or 1-on-1 interviews) to capture responses from enrolled students.

## Conclusions

In an undergraduate seminar course focused on cancer survivorship, an improvement of students’ perceptions and awareness of cancer survivorship-related issues was observed, advocating the value on introducing highly prevalent cancer survivorship topics early to both undergraduate STEM and non-STEM students. Future studies should evaluate whether incorporating experiential learning and additional information would improve knowledge and attitude of cancer survivorship among undergraduates.

## Data Availability

Data is available upon request.
